# Chronic Osteoporotic Pain in Mice: Cutaneous and Deep Musculoskeletal Pain Are Partially Independent of Bone Resorption and Differentially Sensitive to Pharmacological Interventions

**DOI:** 10.1155/2017/7582716

**Published:** 2017-02-19

**Authors:** Miyako Suzuki, Magali Millecamps, Lina Naso, Seiji Ohtori, Chisato Mori, Laura S. Stone

**Affiliations:** ^1^Center for Preventive Medical Sciences, Chiba University, Chiba, Japan; ^2^Faculty of Dentistry, McGill University, 2001 McGill College Avenue, Suite 500, Montreal, QC, Canada H3A 1G1; ^3^The Alan Edwards Centre for Research on Pain, McGill University, 740 Dr. Penfield Avenue, Suite 3200, Montreal, QC, Canada H3G 0G1; ^4^Department of Orthopedic Surgery, Graduate School of Medicine, Chiba University, Chiba, Japan; ^5^Department of Bioenvironmental Medicine, Graduate School of Medicine, Chiba University, Chiba, Japan

## Abstract

Although the pathological changes in osteoporotic bones are well established, the characterization of the osteoporotic pain and its appropriate treatment are not fully elucidated. We investigated the behavioral signs of cutaneous and deep musculoskeletal pain and physical function; time-dependent changes in bone mineral density (BMD) and the emergence of the behavioral phenotype; and the effects of pharmacological interventions having different mechanisms of action (chronic intraperitoneal administration of pamidronate [0.25 mg/kg, 5x/week for 5 weeks] versus acute treatment with intraperitoneal morphine [10 mg/kg] and pregabalin [100 mg/kg]) in a mouse model of ovariectomized or sham-operated mice 6 months following surgery. We observed reduced BMD associated with weight gain, referred cutaneous hypersensitivity, and deep musculoskeletal pain that persisted for 6 months. Chronic bisphosphonate treatment, 6 months after ovariectomy, reversed bone loss and hypersensitivity to cold, but other behavioral indices of osteoporotic pain were unchanged. While the efficacy of acute morphine on cutaneous pain was weak, pregabalin was highly effective; deep musculoskeletal pain was intractable. In conclusion, the reversal of bone loss alone is insufficient to manage pain in chronic osteoporosis. Additional treatments, both pharmacological and nonpharmacological, should be implemented to improve quality of life for osteoporosis patients.

## 1. Introduction

In the USA and Europe, an estimated 30% of postmenopausal women demonstrate clinical signs of osteoporosis [1]. Osteoporosis-related pain often results from local fractures (e.g., vertebrae compression or femoral neck fractures) but is also observed in the absence of bone trauma observable using X-ray, computed tomography (CT), or magnetic resonance imaging examinations. In clinical practice, although 80% of osteoporotic patients complain of low back pain [2], at least 10% do not demonstrate signs of fractures or diseases other than bone demineralization [3]. Hence, the underlying pathophysiological processes involved in osteoporotic pain are not fully understood. Most of our current understanding comes from studies using ovariectomized (OVX) female rodents, which serve as models of postmenopausal women. After removing the ovaries, 2–8 weeks are typically required before significant reductions in* bone mineral density (BMD)* are observed using micro-CT or dual-energy X-ray absorptiometry in mice, rats, sheep, and nonhuman primates [4].

Although deep musculoskeletal pain is the primary concern of patients, most animal studies use cutaneous hypersensitivity to measure pain in osteoporosis models. In ovariectomy-induced rodent models of osteoporosis, cutaneous pain hypersensitivity develops within 4–10 weeks and is dependent on estrogen depletion; estrogen replacement therapy prevents both bone loss and cutaneous pain in the early stages of the pathology [5–9]. Deep musculoskeletal pain is less commonly investigated, with most studies using referred cutaneous hypersensitivity as an indirect measure of deep skeletal pain. As a result, there is a mismatch between the focus of preclinical studies and clinically relevant symptomology.

Pathological changes in osteoporotic bones are well established as contributing to osteoporotic pain. For example, the establishment of acidic microenvironments due to osteoclast hyperactivity might trigger deep bone pain [10] and calcitonin gene-related peptide (CGRP-) immunoreactive pain-sensing nerve fibers increase in osteoporotic bone marrow [11]. Regardless of these advances, the characterization of osteoporotic pain and its appropriate treatment remain to be elucidated. An improved understanding of these issues is crucial for the development of therapeutic strategies to reduce the musculoskeletal pain associated with osteoporosis.

The aims of the present study were to (1) characterize the behavioral signs of cutaneous hypersensitivity, deep musculoskeletal pain, and disability; (2) evaluate time-dependent changes in the emergence of the behavioral phenotype; and (3) examine the effects of pharmacological interventions having different mechanisms of action (chronic administration of pamidronate, a bisphosphonate, versus acute treatment with the gold-standard analgesics, morphine and pregabalin) in a mouse model of ovariectomy-induced osteoporosis, 6 months following surgery.

## 2. Materials and Methods

### 2.1. Animals

Female C57BL6 mice (Charles River Laboratories, Montreal, QC, Canada) were used in all experiments. All mice were housed on a 12 h light/dark cycle in positive-ventilated racks (Allentown, Allentown, NJ), with corncob bedding (7097, Teklad Corncob Bedding, Envigo, Huntingdon, UK) and cotton nesting squares or cardboard huts for enrichment. Mice were given access to food (2092X Global Soy Protein-Free Extruded Rodent Diet, Irradiated, Envigo) and water ad libitum. Before all experimental protocols, animals were habituated to the housing conditions for 1 week.

Mice were randomly assigned to receive either ovariectomies (*n* = 43) or sham surgeries (*n* = 38); all surgical procedures were conducted on 6-week-old animals. Separate groups of mice were evaluated for pain sensitivity at 8 weeks (OVX, *n* = 21; sham-operated, *n* = 10) and 6 months (OVX, *n* = 22; sham-operated, *n* = 28), after surgery. BMDs were evaluated 3 days before behavioral evaluations at both the 8-week and 6-month postsurgical time-points. All experiments were approved by the McGill University Animal Care Committee and conformed to the ethical guidelines established by the Canadian Council on Animal Care and the Committee for Research and Ethical Issues of the International Association for the Study of Pain [12].

### 2.2. Surgical Procedures

A mouse model of OVX-induced osteoporosis was used [4, 8]. The surgical procedure has been described previously [4, 13]. A 1.5 cm dorsal midline incision was made with the cranial terminus 1 cm caudal to the thirteenth rib in a ventral recumbent position under isoflurane anesthesia (flowmeter: 0.4 to 0.8 L/min; isoflurane vaporizer: 2 to 2.5%). Each ovary was ligated and resected after which the uterine horns were placed back into the body cavity, and the muscle wall and skin incisions were closed with 6-0 silk sutures. In the sham-operated group, ovaries were exposed using the identical procedure but were left intact.

### 2.3. BMD

Lumbar vertebrae BMDs were measured at 8 weeks and 6 months, after surgery, using dual-energy X-ray absorptiometry densitometry (GE Lunar PIXImus2 DEXA; Lunar, Madison, WI) following an intraperitoneal (i.p.) injection (0.01 mL/g) of a ketamine/xylazine/acepromazine cocktail (100/20/10 mg/mL, resp.).

### 2.4. Behavioral Procedures

Groups of animals, housed in their home-cages, were habituated to the testing room for 60 min before any manipulation. When relevant, animals were then individually habituated to the testing chamber for 60 min before beginning the behavioral procedures; all testing was conducted between 9:00 a.m. and 3:00 p.m. All behavioral assays were performed as previously described [14–16].

#### 2.4.1. Cutaneous Plantar Sensitivity to Mechanical, Cold, and Heat Stimuli

Mechanical sensitivity was measured using von Frey filaments (Stoelting, Wood Dale, IL) on the plantar surface of the left hindpaw, using an up-and-down technique adapted from Chaplan's method [14, 15, 17]. The von Frey filaments were applied for 4 s or until paw withdrawal, and the 50% threshold for withdrawal (g) was calculated. The stimulus intensity ranged from 0.6 to 4.0 g and corresponded to filament numbers 3.84, 4.08, 4.17, 4.31, and 4.56.

Cold sensitivity was measured using acetone and cold plate tests. In the acetone test, mice were evaluated for cold sensitivity for 1 min after a 25 *µ*L drop of acetone was applied to the plantar surface of the left hindpaw. The total duration of time spent in acetone-evoked behaviors was measured in seconds. Behaviors included paw elevation, flinching, biting, licking, and scratching [14, 15]. In the cold plate test, the latency to the first brisk paw lifting from the 4°C plate (hot/cold plate 35100, Ugo Basile, Varese, Italy) was determined in seconds (cutoff time, 30 s). The assay was repeated 3 times with a >15 min interval between each assessment to avoid cutaneous sensitization. The average of the 3 measurements, for each animal, was used as its individual data.

Heat sensitivity was assessed using the latency of right hindpaw withdrawal from a thermal stimulus, as previously described [18]. Briefly, mice were placed in Plexiglas cages on top of a glass sheet, and a thermal stimulus (IITC Life Science, Woodland Hills, CA) was focused on the center of the plantar surface of the hindpaw. Withdrawal latencies were measured three times at 10 min intervals, and the average was calculated. A cutoff value of 22.7 s was used to prevent tissue damage.

#### 2.4.2. Deep Musculoskeletal Discomfort

The grip test assay was used to assess resistance to anteroposterior stretching. During the grip test assay, the animal was gently stretched, while allowing it to grip a bar with its forepaws, until the point of release; the grip strength at the time of release was expressed in grams [19, 20].

#### 2.4.3. Physical Function

Motor capacity was evaluated in an open field test, using a transparent open field apparatus (24 × 24 cm) placed in a quiet room. Mice were individually placed into the center of the open field and their spontaneous behavior was videotaped for 5 min to assess general motor activity. The total distance covered during the 5 min test period was analyzed, using ANY-maze software (Stoelting), by an observer blinded to the experimental conditions [14–16].

### 2.5. Drug Treatment

Drugs or a saline vehicle (2 mL/kg, i.p.) was administered to OVX and sham-operated mice. BMD was measured 6 months, after surgery, and acute treatments were started the following week. For the chronic pamidronate experiment, BMDs were measured, again, 1 and 5 weeks after the start of the treatment.

#### 2.5.1. Acute Treatment with Morphine and Pregabalin

Using a crossover experimental protocol, mice (OVX, *n* = 22; sham-operated, *n* = 28; 6 months after surgery) were randomly assigned to receive either pregabalin (100 mg/kg, Pfizer, Groton, CT) or saline, as a vehicle control. A minimum washout period of at least 48 h was included before animals were subsequently injected with either morphine (10 mg/kg, Medisca, Montreal, QC, Canada) or saline. Animals were tested at regular intervals after drug administration for 3-4 h after treatment.

#### 2.5.2. Chronic Treatment with the Bisphosphonate Pamidronate

At 72 h after the last acute treatment, mice (OVX, *n* = 22; sham-operated, *n* = 28; 6 months after surgery) were randomly reassigned to receive chronic treatment with either pamidronate (0.25 mg/kg, i.p., Sigma-Aldrich, St. Louis, MO) or a saline vehicle control. The drug or saline was administered 5 days/week for 5 weeks. Pamidronate belongs to the bisphosphonate class of medications commonly administered to patients with osteoporosis or bone metastasis. BMDs and behavioral signs were measured prior to each daily drug/vehicle administration to avoid acute effects or interactions with the anesthetic cocktail. Although chronic pamidronate treatment was the last intervention tested in this study, it is presented first in the results for clarity.

### 2.6. Data Analyses

All data were plotted as means ± standard error, with *n* indicating the number of mice; *P* < 0.05 was considered statistically significant. For body weights, BMDs, and postovariectomy behavioral endpoints, measurements were analyzed using two-way analysis of variance (ANOVA), followed by Tukey's multiple comparison, at 8 weeks and 6 months, after surgery. For the pharmacological studies, two-way repeated measures ANOVA was conducted with group and time as factors, followed by Tukey's multiple comparisons test. Statistical analyses were performed using GraphPad Prism 6 software (GraphPad Software, San Diego, CA).

## 3. Results

### 3.1. Effect of Ovariectomy on Body Weight and BMD

**Figure 1:**
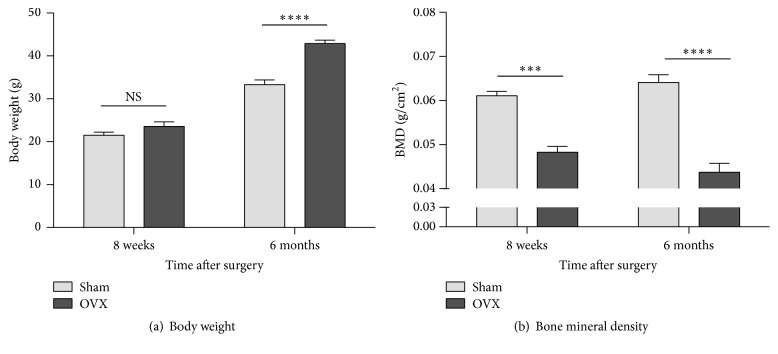
Effect of ovariectomy on body weight and bone mineral density. Body weights are higher in ovariectomized (OVX) mice than in sham-operated (sham) animals at 6 months, but not at 8 weeks, after surgery (a). Vertebral bone mineral density is lower, at both 8 weeks and 6 months after surgery, in OVX mice than in sham mice (b). Data are expressed as means ± standard error of the mean. NS: not significant; ^*∗∗∗*^*P* < 0.001; ^*∗∗∗∗*^*P* < 0.0001; and OVX versus sham, two-way repeated measures analysis of variance (factors = group × time) followed by Tukey's test for multiple comparisons.

Body weight measurements demonstrated that mice in the OVX group were similar in weight to those in the sham-operated group at 8 weeks (not significant [NS]) but were heavier than the sham-operated animals, 6 months after surgery (Figure 1(a)). Densitometry measurements taken from the lumbar vertebrae demonstrated a significantly greater loss of BMD in the OVX group than in the sham-operated group at both 8 weeks and 6 months after surgery, confirming the development and maintenance of osteoporosis for at least 6 months after ovariectomy (Figure 1(b)).

### 3.2. Effects of Ovariectomy on Behavioral Indices of Cutaneous and Deep Musculoskeletal Pain and Physical Function 

#### 3.2.1. Cutaneous Plantar Hypersensitivity

**Figure 2:**
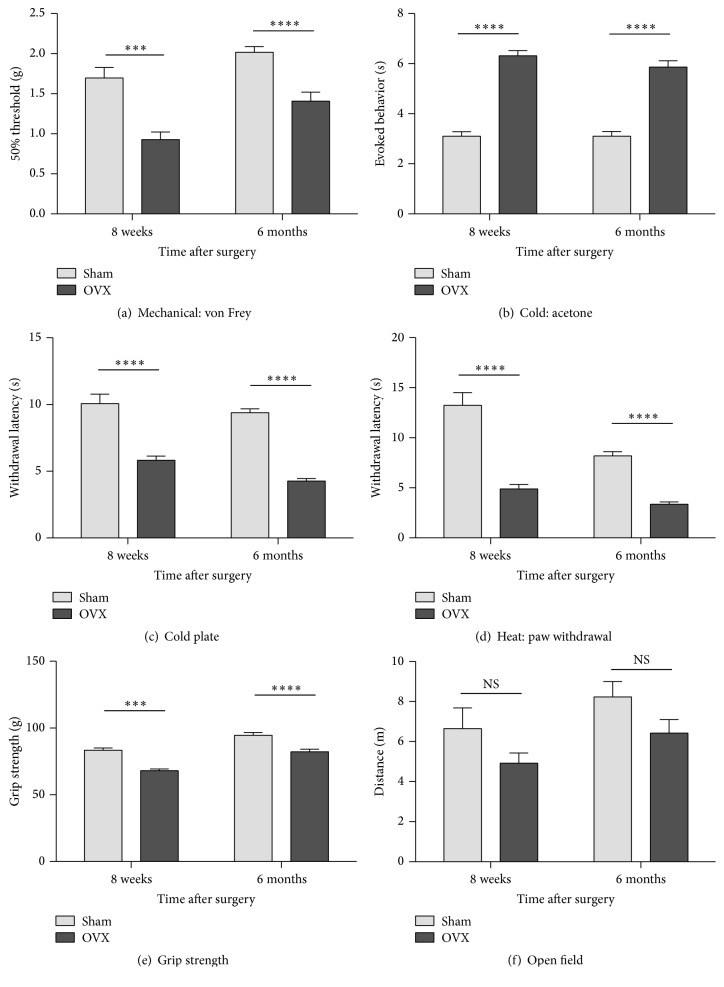
Effects of ovariectomy on behavioral indices of cutaneous and deep musculoskeletal pain and physical function. Hypersensitivity to cutaneous plantar mechanical stimuli (von Frey assay) (a), cold (acetone test) (b) and cold plate tests (c), and heat (radiant heat hindpaw withdrawal assay) (d) develops by 8 weeks and persists for 6 months after ovariectomy. Using the grip force strength assay as a behavioral index of deep musculoskeletal pain, reduced strength is observed at 8 weeks and 6 months, after surgery (e). The total exploration distance during 5-minute exposures to an open field test was used as a behavioral index of physical function; a significant difference is not evident between OVX and sham-operated mice at either time-point (f). Data are expressed as means ± standard error of the mean. OVX: ovariectomized; NS: not significant; ^*∗∗∗*^*P* < 0.001; ^*∗∗∗∗*^*P* < 0.0001; and OVX versus sham-operated, two-way repeated measures analysis of variance (factors = group × time) followed by Tukey's test for multiple comparisons.

Compared to sham controls, ovariectomy resulted in plantar hypersensitivity to mechanical, cold, and heat stimuli, 8 weeks after surgery, and the hypersensitivity persisted for at least 6 months after ovariectomy (Figure 2). Specifically, OVX mice were hypersensitive to mechanical stimuli at 8 weeks and 6 months, after surgery (Figure 2(a)). Increased sensitivity to cold stimuli was also observed following OVX. During the acetone test, the total time engaged in nocifensive behaviors increased in the OVX, compared to the sham-operated, groups at 8 weeks and 6 months after surgery (Figure 2(b)). Similarly, the latency to the first, brisk hindpaw withdrawal from the cold plate was significantly shorter in the OVX group than in the sham-operated group at 8 weeks and 6 months (Figure 2(c)). Heat sensitivity increased in the OVX, compared to the sham-operated groups, as indicated by significantly lower withdrawal latencies at 8 weeks and 6 months, after surgery (Figure 2(d)).

#### 3.2.2. Deep Musculoskeletal Discomfort and Physical Function

Resistance to anteroposterior stretching was assessed using the grip force assay. This assay is used to evaluate deep musculoskeletal pain [20] in murine models of bone cancer pain [21], low back pain [22] and muscle inflammation [23]. In this study, grip strength was significantly reduced in OVX, compared to sham-operated, mice 8 weeks after surgery, and persisted for at least 6 months after surgery (Figure 2(e)). In the open field test, significant changes in exploratory activity were not observed, as measured by the distance travelled, in meters, at either (8-week or 6-month) time-point (Figure 2(f)).

### 3.3. Efficacy of Chronic Bisphosphonate Treatment on Chronic Osteoporosis-Related Cutaneous and Musculoskeletal Pain 6 Months following OVX

Bisphosphonates are first-line treatments for patients with osteoporosis, reversing bone loss and attenuating the associated pain [3]. Chronic bisphosphonate administration also reverses bone loss and reduces cutaneous referred pain in the early stages of the pathology (i.e., 6–8 weeks after ovariectomy) in rodent models of ovariectomy-induced osteoporosis [5]. Therefore, we tested the efficacy of bisphosphonate therapy in mice with chronic, fully established osteoporosis, 6 months after ovariectomy. Mice were treated with the bisphosphonate pamidronate for 5 weeks, starting 6 months after surgery; changes in vertebral BMDs, behavioral signs of cutaneous and deep musculoskeletal pain, and physical functioning were monitored after 1 and 5 weeks of treatment.

**Figure 3:**
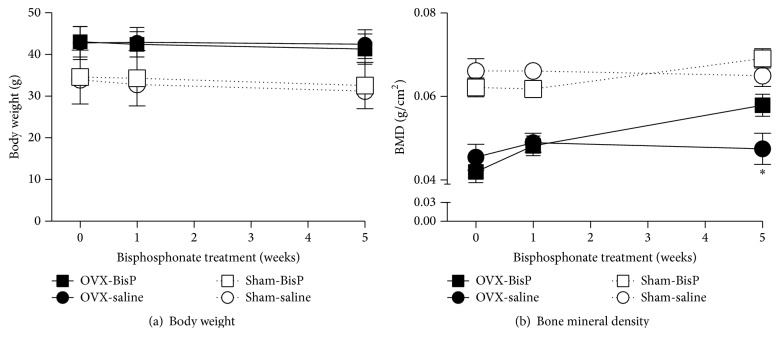
Efficacy of chronic bisphosphonate (pamidronate) treatment on body weight and bone mineral density. Mice were treated with pamidronate (0.25 mg/kg) or a saline vehicle 5 days/week for 5 weeks, beginning 6 months after undergoing ovariectomies or sham surgeries. Pamidronate has no effect on body weight after either 1 or 5 weeks of treatment (a) but results in a significant reversal of ovariectomy-induced bone mineral density decreases after 5 weeks of treatment that is not observed after 1 week of treatment (b). Data are expressed as means ± standard error of the mean. OVX: ovariectomized; BisP: bisphosphonate (pamidronate); ^*∗*^*P* < 0.05; and OVX with bisphosphonate pamidronate versus saline vehicle control, two-way repeated measures analysis of variance (factors = group × time) followed by Tukey's test for multiple comparisons.

**Figure 4:**
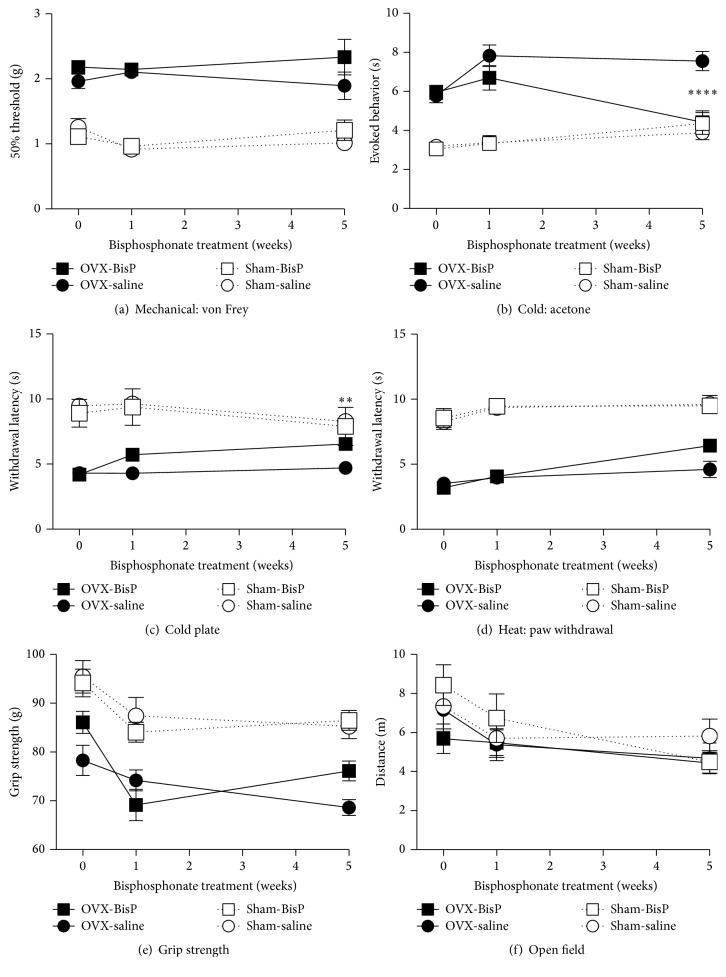
Efficacy of chronic bisphosphonate (pamidronate) treatment on chronic osteoporosis-related cutaneous and musculoskeletal pain and physical function. Pamidronate (0.25 mg/kg) was administered 5 days/week for 5 weeks, beginning 6 months after ovariectomies or sham surgeries. Pamidronate has no effect on cutaneous plantar hypersensitivity to mechanical stimuli (von Frey assay) (a) and reversed hypersensitivity to cold (acetone test) (b) and cold plate tests (c); it was ineffective against heat sensitivity in the radiant heat paw withdrawal assay (d). Pamidronate is also inactive in the grip strength assay (e) and open field test (f). Data are expressed as means ± standard error of the mean. OVX: ovariectomized; BisP: bisphosphonate; ^*∗∗*^*P* < 0.01; ^*∗∗∗∗*^*P* < 0.0001; and OVX group with pamidronate versus saline vehicle control, two-way analysis of variance (factors = group × time) followed by Tukey's test for multiple comparisons.

In sham-operated mice, repeated treatment with pamidronate (0.25 mg/kg, i.p.) for 5 weeks had no effect on vertebral BMD, cutaneous or deep pain, or physical functioning; no body weight effects were observed in either the OVX or sham-operated mice (Figures 3 and 4). In 6-month postovariectomy mice, pamidronate (0.25 mg/kg, i.p.) had no effect on any of these measures after 1 week of repeated treatment. In contrast, when the treatment period was extended to 5 weeks, the reduction in BMD, due to ovary removal 6 months earlier, had been reversed (Figure 3(b)). Five weeks of pamidronate treatment resulted in a significant decrease in cold hypersensitivity, as determined using the acetone and cold plate tests (Figures 4(b) and 4(c)), but had no effect on mechanical or heat sensitivity, grip strength, or open field exploration results (Figures 4(a) and 4(d)–4(f)).

### 3.4. Efficacy of Acute Morphine and Pregabalin Treatment on Chronic Osteoporosis-Related Cutaneous and Musculoskeletal Pain 6 Months following Ovariectomy

Analgesic options typically proposed to osteoporotic patients include nonsteroidal anti-inflammatory drugs (NSAIDs), acetaminophen, and opioids [24]. In the present study, we assessed the acute analgesic efficacy of morphine and pregabalin (the gold-standard treatments for nociceptive/inflammatory and neuropathic pain, resp.) in chronic osteoporosis-induced pain.

#### 3.4.1. Morphine

**Figure 5:**
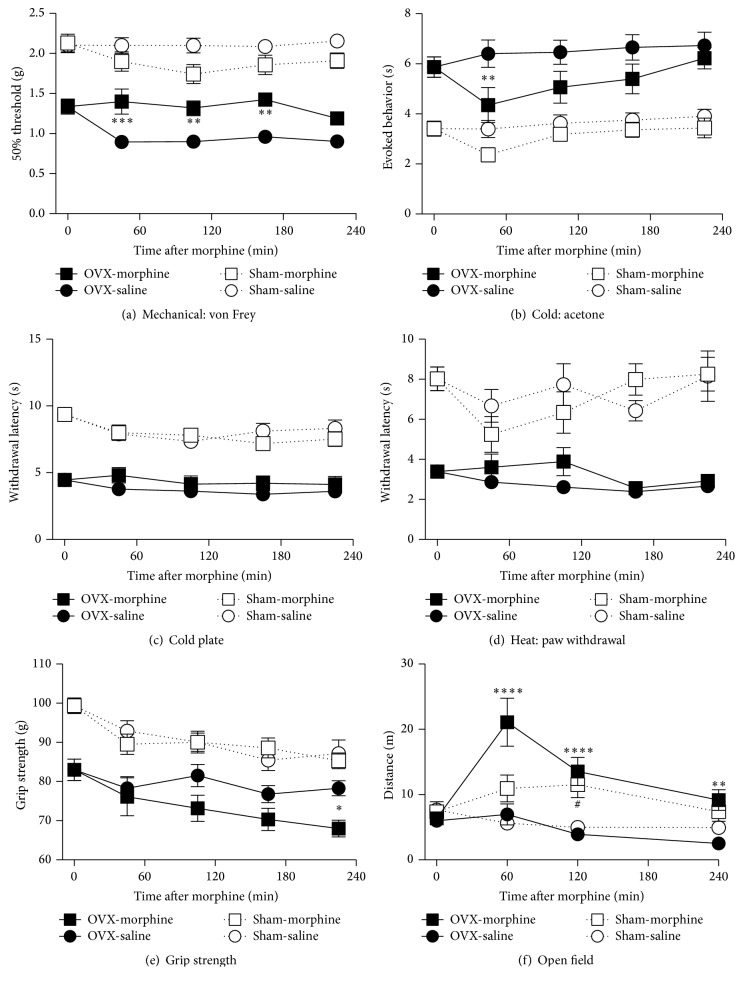
Efficacy of acute morphine treatment on chronic osteoporosis-related cutaneous and musculoskeletal pain and physical function. The effect of an acute intraperitoneal injection of morphine (10 mg/kg) was measured for several hours in mice, 6 months after ovariectomies or sham surgeries. In ovariectomized (OVX) mice, morphine results in a significant reduction in mechanical hypersensitivity (von Frey assay) that persists for several hours (a) and reduces cold hypersensitivity (acetone test) (b). No effects are evident in the cold plate (c), radiant heat paw withdrawal (d), or grip strength (e) assays. Morphine increases the OVX animals' overall activity in the open field test (f). No effects are seen in the sham-operated mice. Data are expressed as means ± standard error of the mean. ^*∗*^*P* < 0.05; ^*∗∗*^*P* < 0.01; ^*∗∗∗*^*P* < 0.001; ^*∗∗∗∗*^*P* < 0.0001; OVX group with morphine versus vehicle, ^#^*P* < 0.05; and sham-operated group with morphine versus vehicle, two-way repeated measures analysis of variance (factors = group × time), followed by Tukey's test for multiple comparisons.

Acute morphine (10 mg/kg, i.p.) administration partially reduced the cutaneous hypersensitivity observed on the hindpaw plantar surface. In osteoporotic mice, hypersensitivity to mechanical stimuli was reduced for 180 min after acute administration of morphine, and hypersensitivity to acetone-evoked cooling was transiently reduced for 60 min, after injection (Figures 5(a) and 5(b)). No analgesic effects of morphine were detected past these time-points or in the cold plate or radiant heat assays (Figures 5(c) and 5(d)). Morphine did not alter hindpaw sensitivity to mechanical, cold, or heat stimuli in sham-operated mice.

In the grip strength test for deep musculoskeletal discomfort, morphine had no analgesic effects (i.e., increase in grip strength) in either the OVX or sham-operated animals, at 3 hours after injection. However, a small decrease in grip strength was observed nearly 4 hours after morphine injection (Figure 5(e)). In the open field test, acute morphine administration resulted in a significant increase in overall activity in both the OVX and sham-operated mice (Figure 5(f)).

#### 3.4.2. Pregabalin

**Figure 6:**
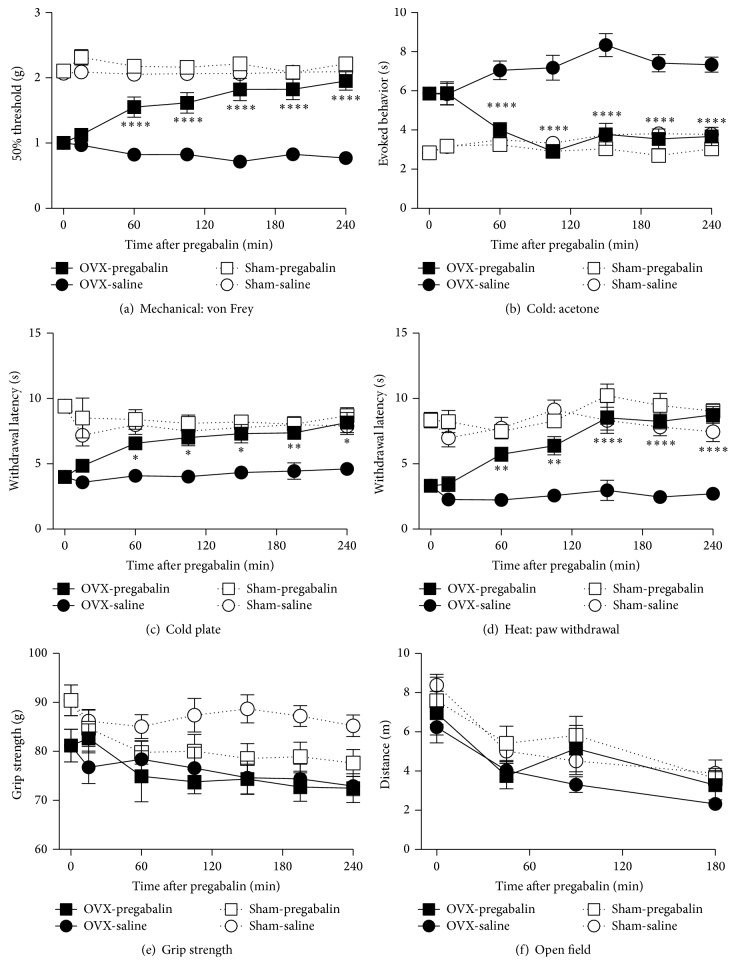
Efficacy of acute pregabalin treatment on chronic osteoporosis-related cutaneous and musculoskeletal pain and physical function. The effects of acute intraperitoneal pregabalin (100 mg/kg) injections were measured for several hours in mice, 6 months following ovariectomies or sham surgeries. Pregabalin significantly reverses cutaneous plantar hypersensitivity to mechanical stimuli (von Frey assay) (a) and cold (acetone test) (b) and cold plate (c) tests and to heat (radiant heat paw withdrawal assay) (d) in OVX, but not sham-operated, mice. Pregabalin had no effect on OVX-operated mice compared to saline but did result in a trend towards decreased grip strength in sham-operated mice (e). No effects were observed in the open field assay (f). Data are expressed as means ± standard error of the mean. OVX: ovariectomized; ^*∗*^*P* < 0.05; ^*∗∗*^*P* < 0.01; ^*∗∗∗∗*^*P* < 0.0001; and OVX group with pregabalin versus vehicle, two-way repeated measures analysis of variance (factors = group × time), followed by Tukey's test for multiple comparisons.

In contrast to morphine, acute pregabalin (100 mg/kg, i.p.) treatment significantly attenuated cutaneous plantar hypersensitivity to mechanical, cold, and heat stimuli in OVX mice, 6 months after surgery, which lasted at least 4 h after the initial administration (Figures 6(a)–6(d)). Pregabalin had no effect on sham-operated animals in those assays.

Pregabalin demonstrated no effect in OVX mice but caused a trend towards reduced grip strength in sham-operated mice suggestive of increased musculoskeletal discomfort (Figure 6(e)). Pregabalin had no effect in either OVX- or sham-operated mice in the open field test (Figure 6(f)).

## 4. Discussion

In this study, we demonstrated reduced vertebral bone density associated with weight gain, referred cutaneous hindpaw hypersensitivity, and deep musculoskeletal pain 8 weeks after ovariectomy which persisted for 6 months. Chronic bisphosphonate treatment for 5 weeks reversed bone loss and hypersensitivity to cold, but most behavioral indices of osteoporotic pain remained unchanged. Although the efficacy of acute morphine on cutaneous pain was weak, pregabalin was highly effective. Deep musculoskeletal pain, as assessed by grip strength, was intractable (i.e., none of the treatments were effective in this assay).

### 4.1. Clinically Relevant Features of the OVX Mouse Model of Osteoporosis: Increased Body Mass and Decreased Bone Density

Ovariectomy results in estrogen deficiency, which is associated with increased body weight, in menopausal women [25]. Previous mouse studies have demonstrated increased weight within the 5 weeks following ovariectomy, a period comparable to 2.5 years of human life [26–28]. In the current study, significant increases in body weight were observed in OVX mice at 6 months, but not 8 weeks, following surgery. The progressive weight gain is consistent with the clinically observed effects of hormone deficiency in menopausal women.

Bone densitometry measurements, obtained 8 weeks and 6 months postoperatively, detected reduced BMD levels in OVX mice, compared with sham-operated mice. Previous studies have similarly supported the validity of this model for studying osteoporosis, demonstrating that by 2 months, after ovariectomy, mice display the hallmark features of osteoporosis [29–31].

The phenotype of the OVX mouse model used in this study includes the clinically relevant features of osteoporosis (weight gain and reduced BMD), consistent with its utility as an osteoporosis model.

### 4.2. Behavioral Indices of Chronic Osteoporotic Pain

In the current study, we observed significant pain hypersensitivity in mice at 8 weeks and 6 months in OVX, compared with sham-operated, mice. One important observation in this study was that the decreased BMD and significant pain hypersensitivity were maintained for 6 months in OVX mice. Only a few previous studies have followed the long-term course of pain sensitivity in OVX rats [32] or mice [5, 33, 34].

#### 4.2.1. Behavioral Indices of Cutaneous Plantar Hypersensitivity

Ovariectomy-induced osteoporotic mice displayed significant hindpaw hypersensitivity to mechanical, cold, and heat stimuli at 8 weeks and 6 months following surgery. Although previous studies have investigated a range of pain-related outcomes, including acute [35, 36] (tail flick and hot plate), tonic (visceral distension and formalin test) [6, 7, 9, 37–40], and neuropathic pain [37] in OVX animals, the conclusions of these studies differ depending on the assessment modality used [41]. For example, most studies of OVX rats reported little or no change in thermal sensitivity [39, 40]. Similarly, studies in mice have reported a lack of hindpaw and tail heat hypersensitivity, even in the presence of mechanical hyperalgesia and allodynia [6, 42]. In contrast, we observed significant heat hypersensitivity in OVX mice at 8 weeks and that hypersensitivity persisted for the full 6-month study period. These inconsistencies may be due to differences in the postsurgery follow-up periods across the studies. In fact, whereas heat hypersensitivity is observed in ovariectomy-induced osteoporotic mice, it has a slow onset, taking approximately 4-5 weeks to develop, postoperatively [5, 7, 9]. Additional explanations include genetically determined sensitivity to each stimulus modality across strains and species or different environmental factors, such as the type of diet or bedding used in the animal housing facility; examples of the aforementioned factors impacting pain sensitivity exist [43–45]. In the current study, we focused on the 8-week and 6-month postinjury time-points to model the full effects of long-term osteoporosis. Although we cannot be sure when, during the first 8 weeks, each symptom emerged, the differences across studies highlight the importance of evaluating multiple sensory modalities and employing appropriately chronic endpoints to fully characterize a model.

#### 4.2.2. Behavioral Indices of Deep Musculoskeletal Pain and Physical Function

The grip strength reduction in the OVX group, at 8 weeks and 6 months following ovariectomy, suggests the presence of deep musculoskeletal discomfort not previously reported in this model. The grip strength reduction may be related to multiple factors, including muscle weakness or skeletal pain. Bone and muscle tissues have a close relationship with one another, and aging contributes to loss of functionality in both. In humans, muscle weakness is related to a progressive decline in bone mass, with consequent axial kyphosis, skeletal deformities, joint imbalances, and tension in muscular structures [46].

The most common symptom in patients with osteoporosis is severe or intolerable back pain [24]. In mouse models of low back pain associated with intervertebral disc degeneration, we have shown that mice display axial discomfort, characterized by a decreased grip strength similar to that observed in the OVX mice in the current study [15, 22, 47]. Therefore, the decreased grip strength observed here could be a behavioral sign of low back pain. Unfortunately, based on current data, concluding whether the underlying source is vertebral bone, intervertebral disc, muscle, or another structure is difficult. The insensitivity of this phenotype to either chronic bisphosphonate or acute analgesia (morphine and pregabalin) does not provide additional insight, and other studies have not evaluated axial or deep musculoskeletal pain derived from an animal model of osteoporosis. We propose, however, that the grip strength reduction is not likely due to overall weakness, as the OVX animals performed normally in the open field test. In addition, ovariectomy-induced osteoporotic mice have been reported to display decreased physical function in the rotarod and treadmill assays, compared with naive controls [5, 11]. This suggests that the primary behavioral phenotype in osteoporotic mice is pain hypersensitivity, rather than decreased physical functioning.

### 4.3. Pharmacological Treatment for Pain in Osteoporotic Mice: Efficacy of the Bisphosphonate Pamidronate

Pamidronate belongs to the bisphosphonate class of medications that exerts a restorative effect on BMD [3, 48]. Bisphosphonates are potent medications that reestablish a balance between bone resorption and formation by acting on osteoclasts; they are generally used in patients with osteoporosis [49–51]. In the current study, chronic pamidronate treatment resulted in reversal of BMD loss and cold hypersensitivity but had no effect on mechanical or cutaneous heat hypersensitivity, deep musculoskeletal discomfort, or overall physical activity.

The analgesic effects of bisphosphonate have been previously reported, and clinical research has demonstrated their analgesic effects when given to osteoporotic patients with diffuse low back pain [3, 52, 53]. In preclinical studies, complete Freund adjuvant-induced osteoclastic bone resorption and hyperalgesia were significantly suppressed by bisphosphonate treatment [10]. In addition, bisphosphonates reduced ongoing and movement-evoked bone cancer pain, bone destruction, and pathological sensory innervation into the bone, in mice [54]. In ovariectomy-induced osteoporotic rodent models, bisphosphonates cause increased pain thresholds [5] and suppress pain-related sensory neuron activity, in vitro [11]. As previously suggested, improvements in osteoporosis-related pain behavior in bisphosphonate-treated OVX mice may be due to reduced osteoclast activity. In the current study, chronic bisphosphonate treatment clearly reversed ovariectomy-induced BMD loss but demonstrated very weak analgesic effects in animals with chronic osteoporotic pain. Consequently, these findings suggest that chronic osteoporotic pain becomes independent of the initiating disease or pathology. This transition from an acute somatic disorder to chronic pain may explain why pain often persists after the initial mechanical source of the pain has been treated.

### 4.4. Acute Pharmacological Treatment for Pain in Osteoporotic Mice: Efficacy of Morphine and Pregabalin

Morphine treatment (10 mg/kg, i.p. injection) showed partial efficacy against mechanical and cold hypersensitivity but was ineffective for alleviating other behavioral signs of osteoporosis-related pain, 6 months following ovariectomy. Morphine also increased exploratory activity in the open field assay. Since morphine generally produces increased activity, these findings reflect a normal morphine effect [55–58]. In addition, the open field assay hyperactivity demonstrates that the dose of morphine was sufficiently high to produce behavioral changes. Therefore, the weak efficacy observed in the pain-related measures was unlikely due to an insufficient dose. In general, morphine is effective for alleviating nociceptive and inflammatory pain [59] and is widely used in clinical practice because of its effectiveness against many types of pain, including bone pain derived from metastatic bone tumors [60, 61]. However, in our study, morphine had a small effect on the chronic pain associated with ovariectomy-induced osteoporosis. What are the possible causes for this observation? There may be a role for serotonin (5-HT) receptors on *γ*-aminobutyric acid (GABA) neurons. The 5-HT receptors have been previously reported to be downregulated in the descending inhibitory pathway of ovariectomy-induced osteoporotic mice [38]. Dysfunction of the descending inhibitory pathways, perhaps including downregulation of 5-HT receptors on GABAergic neurons, may contribute to the limited efficacy of morphine in chronic osteoporotic pain. Thus, the mechanisms driving chronic osteoporotic pain may be insensitive to morphine.

In contrast to morphine, pregabalin (100 mg/kg, i.p. injection) significantly attenuated mechanical, cold, and heat hypersensitivity in OVX mice, in the current study. Pregabalin is a first-line treatment for neuropathic pain and is highly effective against neuropathic pain symptoms in many animal models [62–64]. Pregabalin is a drug that targets the calcium channel subunit (2) delta ligands, which are particularly localized at synapses, resulting in decreased excitability in presynaptic terminals, thereby reducing neurotransmitter release and central sensitization [65, 66]. The calcium channel subunit alpha (2) delta-1 is upregulated in dorsal root ganglion neurons in animal models of neuropathic pain, consistent with a peripheral component to its mechanism of action [65].

In the grip strength assay pregabalin had no effect in OVX mice but caused a trend towards reduced grip strength in sham-operated mice, suggestive of increased musculoskeletal discomfort. Although weakness is a side-effect of pregabalin, it is not commonly reported. Thus, the reduced muscle strength in pregabalin-treated sham animals is unlikely to represent a serious adverse effect. Although many mechanisms contribute to musculoskeletal pain, in general it is reported to be more inflammatory than neuropathic [67]. This may explain why, in the current study, pregabalin attenuated OVX-induced hypersensitivity in the hindpaw but had no effect OVX-induced deep musculoskeletal pain.

Antiepileptic mediation is not typically given to osteoporotic patients for pain management. Although these drugs are efficacious in many painful musculoskeletal conditions [68], their deleterious interactions with vitamin D and calcium metabolism have prevented their use in osteoporosis. However, new generations of anticonvulsants are safer, especially when associated with vitamin D and calcium supplementation [69] and aging epileptic patients that develop osteoporosis, now have excellent outcomes.

This is the first report to show that pregabalin has analgesic efficacy in the treatment of chronic osteoporotic pain, in mice, suggesting that chronic osteoporotic pain is associated with sensitization of the peripheral and central nervous systems. Although NSAIDs are widely used as general analgesics, harmful effects on bone metabolism in patients with chronic pain and osteoporosis have been reported, questioning their broad utility [24, 70–72]. Moreover, morphine inhibits bone healing and exacerbates the progression of bone cancer [73, 74]. In contrast, to date, there are no reports of pregabalin having adverse effects on bone metabolism. Therefore, these findings suggest that pregabalin might be clinically useful for osteoporosis-related pain that is resistant to other analgesics, such as opioids or NSAIDs, or when their use is contraindicated.

### 4.5. Pain in Osteoporotic Mice: Establishment and Maintenance

#### 4.5.1. Estrogen

Although estrogen receptors have been demonstrated to be downregulated and steroid hormones are gradually decreased in mature OVX rats [75], estrogen replacement therapy has no long-term analgesic effect in these animals [32, 76, 77]. Furthermore, clinical studies have reported that estrogen replacement has no analgesic effect and is not recommended for osteoporotic patients with chronic pain [78–81]. These findings are consistent with the use of OVX, rather than estrogen-deficient, mice as a model of osteoporotic pain.

#### 4.5.2. Acid and Innervation

Deep bone pain is less well studied, behaviorally, than cutaneous inflammatory or neuropathic pain, but it is believed that the establishment of acidic microenvironments in osteoporotic bones, due to osteoclast hyperactivity, might trigger this pain [10]. Such pH decreases likely stimulate acid-sensing receptors, including the acid-sensing ion channel and the local transient receptor potential vanilloid-1 [52, 82, 83], and increases production and secretion of proinflammatory cytokines in osteoporotic bones [10, 84, 85]. Furthermore, CGRP-immunoreactive pain-related sensory nerve fibers increase in osteoporotic bone marrows [11]. These acidic and innervation changes would affect sensory input, creating a local neurogenic inflammatory state and osteoporotic pain.

#### 4.5.3. Vitamin D

The vitamin D receptor has been observed in skeletal muscle [86] and gradually decreases in density with aging or osteoporosis, followed by muscle atrophy and fat generation [87]. Moreover, osteoporosis has previously been reported to be associated with sarcopenia accompanied by muscle atrophy [88]. These changes have been reported to cause musculoskeletal pain [88, 89]. Thus, osteoporotic pain might be affected by vitamin D changes, which also provides a possible explanation for why deep musculoskeletal pain, in this study, was resistant to bisphosphonates. Further studies evaluating the relationship between osteoporotic pain and vitamin D are needed to better understand the mechanisms underlying different pain symptoms experienced by osteoporotic patients.

#### 4.5.4. Central Sensitization

In OVX mice, some reports have shown results consistent with central sensitization. In OVX mice, 8 weeks after surgery, increased numbers of c-Fos-immunoreactive neurons, a marker of neurogenic activity, were observed in laminae I-II of the spinal cord dorsal horn [5]. In addition, the long-term effects of ovariectomy (at 6 or 22 months after surgery) include age-dependent changes in the morphology of hippocampal astroglia and microglia, suggestive of neuroinflammation and neuroplasticity [90]. Central neuroplasticity is a well-established consequence of chronic pain. Here, we demonstrated that ovariectomy-induced chronic pain persisted 6 months after ovariectomy; therefore, central sensitization likely plays an important role in chronic osteoporotic pain.

### 4.6. Future Directions

We demonstrated that osteoporosis-related cutaneous and deep musculoskeletal pain, 6 months after ovariectomy, becomes chronic. This study is the first to demonstrate that, in a preclinical model, although bone loss can be reversed by bisphosphonate at this chronic stage, the associated pain was difficult to reduce. This suggests that very chronic osteoporotic pain involves mechanisms other than bone mineral loss. Examples of the potential mechanisms that require further exploration include the roles of osteoclast activity, vertebral microfractures, pathological osteoporotic bone innervation, vitamin D receptors, neuropathy, and central nervous system plasticity.

Acute treatment with pregabalin, but not morphine, was highly effective against referred cutaneous hindpaw hypersensitivity. Since we examined only one dose of each drug, our ability to make conclusions regarding overall efficacy is limited. Regardless, these results suggest that pregabalin should be considered for patients with osteoporosis-related pain, if bisphosphonates are insufficient. In addition to characterizing the efficacy of established drugs, new drugs (e.g., antinerve growth factor antibodies) and nonpharmacological, lifestyle-based interventions (e.g., diet, exercise therapy) could be tested using this model. These studies would help develop new strategies for the treatment of chronic osteoporotic pain.

To conclude, this study demonstrates that the reversal of bone loss alone is insufficient to manage pain in chronic osteoporosis. Additional treatments, both pharmacological and nonpharmacological, should be implemented to improve quality of life for those affected by this disease.
